# Laterality and Sex Differences of Human Lateral Habenula Afferent and Efferent Fiber Tracts

**DOI:** 10.3389/fnins.2022.837624

**Published:** 2022-06-16

**Authors:** Frederick L. Hitti, Drew Parker, Andrew I. Yang, Steven Brem, Ragini Verma

**Affiliations:** ^1^Department of Neurosurgery, University of Pennsylvania, Philadelphia, PA, United States; ^2^Diffusion and Connectomics in Precision Healthcare Research Lab, Department of Radiology, University of Pennsylvania, Philadelphia, PA, United States

**Keywords:** probabilistic tractography, lateral habenula, depression, human brain asymmetry, stria medullaris, fasciculus retroflexus, sex differences

## Abstract

**Introduction:**

The lateral habenula (LHb) is an epithalamic nucleus associated with negative valence and affective disorders. It receives input *via* the stria medullaris (SM) and sends output *via* the fasciculus retroflexus (FR). Here, we use tractography to reconstruct and characterize this pathway.

**Methods:**

Multi-shell human diffusion magnetic resonance imaging (dMRI) data was obtained from the human connectome project (HCP) (*n* = 20, 10 males) and from healthy controls (*n* = 10, 6 males) scanned at our institution. We generated LHb afferents and efferents using probabilistic tractography by selecting the pallidum as the seed region and the ventral tegmental area as the output target.

**Results:**

We were able to reconstruct the intended streamlines in all individuals from the HCP dataset and our dataset. Our technique also aided in identification of the LHb. In right-handed individuals, the streamlines were significantly more numerous in the left hemisphere (mean ratio 1.59 ± 0.09, *p* = 0.04). In left-handed individuals, there was no hemispheric asymmetry on average (mean ratio 1.00 ± 0.09, *p* = 1.0). Additionally, these streamlines were significantly more numerous in females than in males (619.9 ± 159.7 vs. 225.9 ± 66.03, *p* = 0.04).

**Conclusion:**

We developed a method to reconstruct the SM and FR without manual identification of the LHb. This technique enables targeting of these fiber tracts as well as the LHb. Furthermore, we have demonstrated that there are sex and hemispheric differences in streamline number. These findings may have therapeutic implications and warrant further investigation.

## Introduction

The lateral habenula (LHb) is a well-conserved epithalamic nucleus that participates in value-based decision-making (Hikosaka, 2010). LHb neurons are more active during punishment or following the absence of an expected reward. In this manner, LHb activity signals the negative valence associated with a decision (Hikosaka et al., 2008; Hikosaka, 2010; Metzger et al., 2017). Moreover, increased activity of the LHb is seen in response to chronic stress (Hikosaka, 2010; Nuno-Perez et al., 2018). In humans, positron emission tomography has been used to demonstrate that LHb activity is associated with depressed mood (Morris et al., 1999). In rodents, inhibition of the LHb has been shown to reverse social withdrawal and despair seen in animals subjected to chronic stress (Winter et al., 2011; Sachs et al., 2015; Tchenio et al., 2017). Finally, modulation of the LHb with DBS has been demonstrated to be an effective therapy in a patient with treatment resistant depression (TRD) (Sartorius et al., 2010). For these reasons, the LHb may be an important therapeutic target for depression.

To employ neuromodulation of the LHb for therapeutic purposes, its anatomy in humans must be characterized and targeted. The LHb receives input from a number of brain regions *via* the stria medullaris (SM). Predominant inputs include the ventral pallidum and the globus pallidus internus (GPi) which is homologous to the rodent entopeduncular nucleus (Knowland et al., 2017; Hu et al., 2020; Liu et al., 2020). Prior studies in the social defeat mouse model of depression have also demonstrated that these inputs mediate depression-like behavior (Knowland et al., 2017; Liu et al., 2020). Furthermore, the LHb serves as a regulator of the dopaminergic system *via* the fasciculus retroflexus (FR) which connects the LHb to the ventral tegmental area (VTA) (Wise, 2004; Hikosaka et al., 2008). To characterize these fiber tracts in human subjects, we utilized diffusion magnetic resonance imaging (dMRI). Prior studies have reconstructed the SM with dMRI using the LHb as a seed region for probabilistic tractography (Strotmann et al., 2014; Kochanski et al., 2016, 2018; Grodd et al., 2020). While effective, this strategy does not allow for selection of specific input and output tracts that may be associated with depressive symptomatology. Additionally, this strategy requires manual selection of the LHb which can be difficult due to its small size. Here, we selected the pallidum as the seed region and the VTA as the target region to reconstruct the SM and FR. This strategy allowed us to reconstruct these tracts without manual selection of the LHb and enabled characterization of the specific fiber tracts implicated in depression.

## Materials and Methods

### Datasets

Multi-shell 3T dMRI (1.25 mm isotropic resolution, *b* = 1000, 2000, and 3000 s/mm^2^, 90 directions each) and structural MRI (3T T1/T2, 0.7 mm isotropic resolution) data was obtained from the WU-Minn HCP 1200 Subjects Data Release (Van Essen et al., 2012; Glasser et al., 2013). 5 right-handed individuals of each sex (Edinburgh inventory [EI] (Oldfield, 1971) score of 100) and 5 left-handed individuals of each sex (EI score < −80) that contained a complete set of structural and diffusion data were selected. Other than the handedness and data completeness criteria, subject selection was random. To assess the feasibility of our tractography strategy on “clinical grade” data, we selected 10 healthy control individuals (6 males, 4 females) scanned at our institution. This dataset contained 3T T1 structural MRI data (1 mm isotropic resolution) and 3T multi-shell dMRI data (2 mm isotropic resolution, *b* = 300 [15 directions], *b* = 800 [30 directions], and *b* = 2000 [64 directions] s/mm^2^). The number of subjects was selected based on our prior experience with diffusion tractography of small white matter tracts (Yang et al., 2022).

### Probabilistic Tractography

All dMRI data was pre-processed with the “eddy” tool in the FMRIB Software Library (FSL) to correct for eddy current distortions and subject motion and the “topup” tool to correct for EPI distortion (Jenkinson et al., 2012; Andersson and Sotiropoulos, 2016). This data was then further processed using FSL’s bedpostx_gpu followed by probtrackx_gpu for probabilistic tractography (Jenkinson et al., 2012; Hernández et al., 2013; Hernandez-Fernandez et al., 2019). Pre-processed bedpostx_gpu data was available for the HCP dataset. This processing was done with the default settings other than the following parameters: burnin = 3000, zeppelins deconvolution model (Sotiropoulos et al., 2016), gradient non-linearity correction, and Rician noise assumption (Andersson, 2008). For the clinical grade data, the default settings were used for bedpostx_gpu processing except Rician noise was selected (Andersson, 2008; Jbabdi et al., 2012). probtrackx_gpu was used for probabilistic tractography with the ipsilateral pallidum set as the seed region (Supplementary Figure 1). This region of interest (ROI) was generated using FreeSurfer’s subcortical segmentation feature (Fischl, 2012). The VTA was used as the termination mask (Supplementary Figure 2). The VTA ROI was manually drawn as described by Coenen et al. (2012) using T2 structural images (HCP data) or mean b0 images (clinical grade data).

To ensure selection of LHb afferents and efferents, a waypoint mask that contains the LHb was also included. The rostral half of a 6 mm radius sphere centered at the posterior commissure was selected as the waypoint ROI (Supplementary Figure 3). The posterior commissure was chosen as a reference point due to its anatomical proximity to the LHb and ease of identification. To investigate the feasibility of this tractography strategy on single-shell dMRI data, the *b* = 800 data was extracted from the multi-shell clinical grade dataset and processed with bedpostx_gpu and probtrackx_gpu as described above. The *b* = 800 shell was chosen since it was closest to the *b* = 1000 shell used in most clinical studies. For streamline visualization of both the multi-shell and single-shell tractography, the fdt_paths output files from probtrackx_gpu were loaded into FSLeyes, pseudocolored, and overlayed on the structural T1 images. The lower threshold of the fdt_paths display/clipping range was set as 5% of the total number of streamlines generated. To investigate hemispheric differences, the total number of streamlines generated for each hemisphere was compared to the contralateral hemisphere within each subject in the HCP dataset. For sex difference comparisons, the total number of streamlines generated for each hemisphere was summed for each individual in the HCP dataset. The number of generated streamlines was obtained from the waytotal output file.

### Statistical Analysis

Statistical analysis was performed using the GraphPad Prism software. Averages are presented as the mean ± standard deviation (SD) for demographic data and mean ± standard error of the mean (SEM) otherwise. Ratio paired, two-tailed *t*-tests were used to test the significance of hemispheric differences in streamline number. An unpaired, two-tailed *t*-test was used to test for sex differences. Statical significance was defined as *p* < 0.05.

## Results

### Subject Demographics

For the HCP dataset, 10 individuals of each sex, with a full set of dMRI and structural MRI data, were selected. The mean age was 28.7 ± 3.3 years old (range 22–34). Half of the individuals were right-handed (EI score of 100), and half were left-handed (average EI score of −94 ± 6.6, range −100 to −85). The 10 subjects (6 male, 4 female) in our “clinical grade” dataset had an average age of 39.5 ± 8.2 years old (range 26–56).

### Probabilistic Tractography of the Stria Medullaris and Fasciculus Retroflexus

**FIGURE 1:**
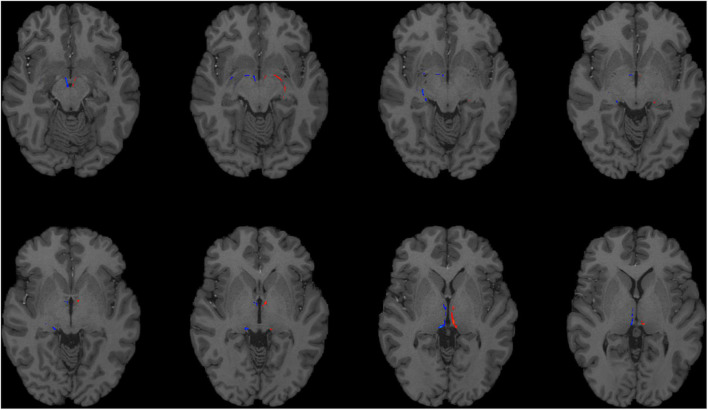
Using HCP dMRI data to reconstruct the afferent and efferent tracts of the LHb. Axial T1 MR images with superimposed streamline density maps of LHb fiber tracts are shown for a representative HCP subject. Left hemisphere streamlines are colored in red and right hemisphere streamlines are colored in blue.

**FIGURE 2:**
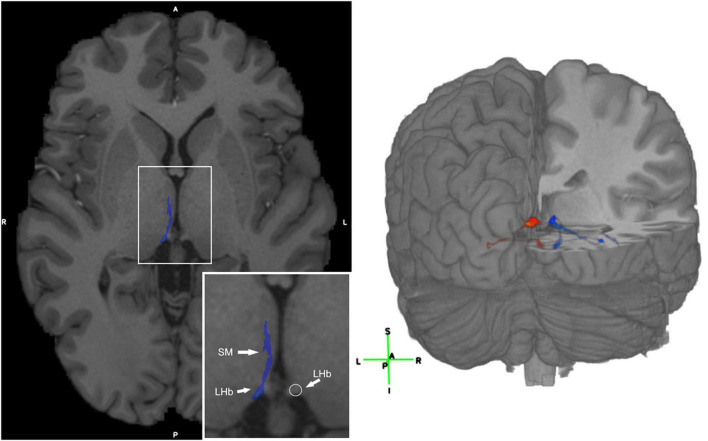
Visualization of the LHb and 3D visualization of the SM and FR streamlines. **(Left)** Axial T1 image with the SM labeled in blue in a representative subject. The LHb is depicted on the image as the area of highest streamline density. **(Right)** 3D visualization of the SM and FR streamlines in a representative HCP subject. Legend: anterior (A), posterior (P), right (R), left (L), superior (S), inferior (I).

**FIGURE 3:**
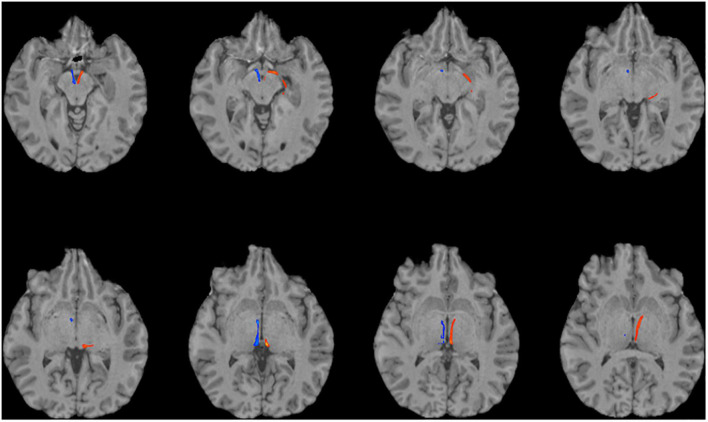
Using multi-shell “clinical grade” dMRI data to reconstruct the afferent and efferent tracts of the LHb. Axial T1 MR images with superimposed streamline density maps of LHb fiber tracts are shown for a representative subject. Left hemisphere streamlines are colored in red and right hemisphere streamlines are colored in blue.

**FIGURE 4:**
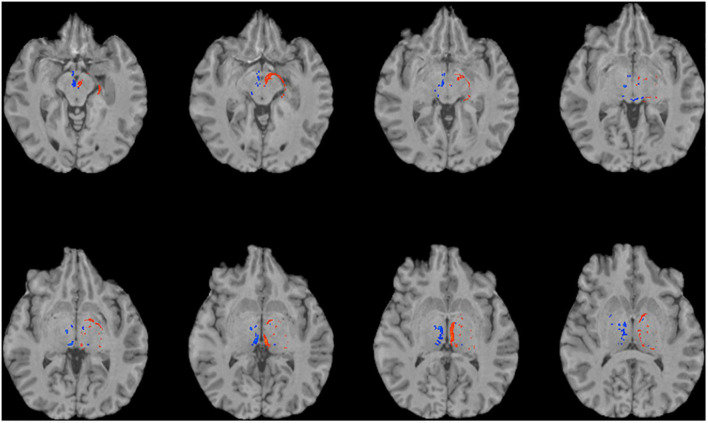
Using single-shell “clinical grade” dMRI data to reconstruct the afferent and efferent tracts of the LHb. Axial T1 MR images with superimposed streamline density maps of LHb fiber tracts are shown for a representative subject. Single-shell/lower *b*-value dMRI does not reconstruct the streamlines as faithfully as the multi-shell/higher *b*-value data. Left hemisphere streamlines are colored in red and right hemisphere streamlines are colored in blue.

To reconstruct the SM and FR, we employed probabilistic tractography using FSL’s bedpostx and probtrackx programs. We used the pallidum as the seed region (Supplementary Figure 1), because the GPi and ventral pallidum project to the LHb *via* the SM. The VTA, a major target of the LHb, was selected as a termination mask to reconstruct the FR (Supplementary Figure 2). A waypoint mask that contained the LHb (but did not require manual outlining of the LHb, see the section “Materials and Methods”) was included to ensure specificity of the tractography (Supplementary Figure 3). Given the relatively small caliber of these tracts, we first investigated the feasibility of this strategy on the high quality dMRI data obtained as part of the HCP. We were able to successfully reconstruct the intended tracts in all HCP subjects (*n* = 20, Figures 1, 2). To explore the generalizability of this strategy and its potential application for clinical purposes, we performed probabilistic tractography on lower resolution “clinical grade” multi-shell dMRI data obtained at our institution. We successfully reconstructed the SM and FR tracts on this data as well in all subjects (*n* = 10, Figure 3). To determine if multi-shell and/or high *b*-value dMRI data is required for the reconstruction of these tracts, we extracted one shell from our multi-shell dataset and employed the tractography strategy detailed above on the single-shell dMRI data. This dMRI data was intended to simulate dMRI data obtained during routine clinical studies. While a number of streamlines were generated, the SM and FR tracts were not reconstructed as robustly using this single-shell and lower *b*-value data (Figure 4).

### Hemispheric Asymmetry of Lateral Habenula Afferents/Efferents

**FIGURE 5:**
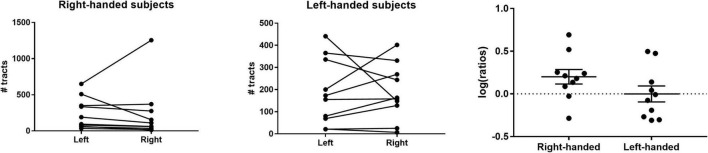
Hemispheric asymmetry of LHb afferents/efferents. **(Left)** The majority (80%) of right-handed individuals had a greater number of streamlines in the left hemisphere. **(Middle)** Left-handed individuals did not demonstrate a consistent hemispheric asymmetry in streamline count. **(Right)** The log of the left:right streamline ratio for each individual is shown along with the group geometric mean. The geometric mean was significantly greater than 0 for right-handed individuals but not for left-handed subjects.

To study possible hemispheric differences in LHb connectivity, we compared the total number of streamlines generated per hemisphere within each subject. We found that 80% of right-handed individuals had a greater number of streamlines in the left hemisphere (Figure 5). In these subjects, there was a significant difference in the mean number of streamlines between hemispheres (geometric mean ratio 1.59 ± 0.09, *p* = 0.04, Figure 5). Conversely, there was no statistically significant hemispheric asymmetry on average in left-handed subjects (geometric mean ratio 1.00 ± 0.09, *p* = 1.0, Figure 5).

### Lateral Habenula Afferent/Efferent Sex Differences

**FIGURE 6:**
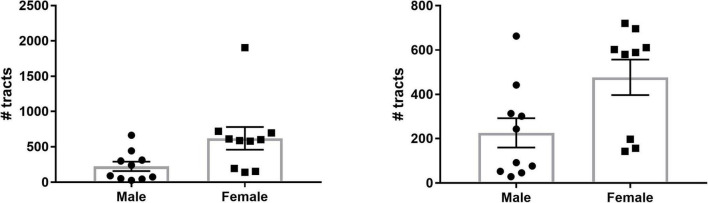
Sex differences in LHb afferent/efferent streamline count. **(Left)** The number of streamlines for each subject as well as the group means are shown. There are a significantly greater number of LHb streamlines in female brains compared to male brains. **(Right)** Even with the outlier removed, the mean number of streamlines in females remains significantly greater than in males.

To investigate potential sex differences of SM/FR streamline count, we summed the total number of streamlines for the two hemispheres of each individual and calculated the group mean. On average, the total number of streamlines was significantly greater in females than in males (619.9 ± 159.7 vs. 225.9 ± 66.03, *p* = 0.04, *n* = 10 for each group, Figure 6). One of the female subjects was an outlier (>2 SD from mean) and the variances of the groups were significantly different (*F* = 5.85, *p* = 0.02). After removal of this subject, the variances of the groups were not significantly different and the means remained significantly different (477 ± 79.71 vs. 225.9 ± 66.03, *p* = 0.03, Figure 6).

## Discussion

In this study, we describe a novel method for reconstruction of the human afferent (SM) and efferent (FR) tracts of the LHb using dMRI data. Prior studies used the LHb itself as a seed region to generate the putative afferent and efferent tracts of this epithalamic nucleus (Strotmann et al., 2014; Kochanski et al., 2016, 2018; Grodd et al., 2020). The LHb is a very small brain region; therefore, manual selection is difficult and may lead to operator dependent variability and inconsistencies in tractography results (Schilling et al., 2021). Our technique employs a seed region automatically generated by FreeSurfer (Fischl, 2012) segmentation, thereby making this method more reliable and operator independent. While the strategy employed here does require manual outlining of the VTA, this region is larger than the LHb and multiple anatomic landmarks exist to identify this region in a consistent manner (Coenen et al., 2012).

Given the LHb’s relevance in affective dysfunction (Hu et al., 2020), targeting this nucleus and/or the SM/FR may be important for therapeutic purposes. The method employed here allows for identification of these tracts for surgical targeting. One limitation of this technique is that the streamlines include both the afferents and efferents, so they are not easily separable. Notably, other previously published techniques have this limitation as well. Furthermore, the LHb itself may be identified as the brain region adjacent to the third ventricle with the highest streamline density (Figure 2). We first used the high quality HCP dMRI data to develop and validate our tractography method and then demonstrated that this method is generalizable to dMRI data that may be acquired in a clinical setting. Hence, this tractography strategy may potentially be used for treatment purposes in the future.

After developing a strategy to generate the SM/FR tracts, we then proceeded to further characterize these tracts. We found hemispheric asymmetry in the number of streamlines generated in a majority of individuals, and this asymmetry was statistically significant in right-handed subjects. The LHb is a well-conserved epithalamic nucleus found in all vertebrates (Bianco and Wilson, 2009). Structural studies in multiple species, including humans, have demonstrated that there are hemispheric differences in the volume of the lateral habenular nucleus (Bianco and Wilson, 2009; Ahumada-Galleguillos et al., 2017). Interestingly in many species of fish, the right LHb is much larger than the left; however, in mammals the hemispheric difference is less pronounced and the left LHb is often larger than the right (Bianco and Wilson, 2009; Ahumada-Galleguillos et al., 2017). To our knowledge, our study is the first to demonstrate in living human subjects that the input and output streamlines of the LHb exhibit hemispheric asymmetry.

In addition to structural asymmetry, several groups have reported differences in LHb function between the two hemispheres (Lawson et al., 2014; Hennigan et al., 2015; Amiri et al., 2021). One group used resting state functional MRI (fMRI) to show that patients with TRD had increased functional connectivity of the left LHb compared to controls (Amiri et al., 2021), while another group demonstrated decreased node degree of the right LHb in individuals with subclinical depression relative to controls (Zhu et al., 2019). Other groups have explored the role of the LHb using fMRI in healthy subjects and have demonstrated that LHb activity is associated with an aversive stimulus (shock) (Lawson et al., 2014; Hennigan et al., 2015). While both studies demonstrated asymmetric LHb activation, Hennigan et al. (2015) reported increased activity of the left LHb compared to the right, but Lawson et al. (2014) reported increased activity of the right LHb, not the left. Lawson and colleagues suggested that they observed right LHb activation because the shock was delivered to the subjects’ left hand (Lawson et al., 2014). In contrast, the Hennigan et al. study reported left LHb activation with shocks delivered to the subjects’ left foot (Hennigan et al., 2015). These authors did not speculate regarding the cause of asymmetric LHb activation, but their results do not support the hypothesis that the location of the noxious stimulus dictates which LHb nucleus is activated. Considered together, the structural and functional data suggest that the left LHb is dominant in most individuals. Neither of the fMRI studies reported the handedness of their subjects. Our structural data suggest that handedness may potentially explain the divergent findings of these studies. Although the streamline count was greater on the left in 80% of right-handed subjects, there were some subjects with a greater streamline count on the right. Asymmetric LHb volume or afferent/efferent streamline number may correlate with functional LHb asymmetry, and future studies could explore the correlation between LHb structure and function within individuals.

Psychological studies have demonstrated that individuals associate positive valence to objects/individuals in the space in which their dominant hand acts more easily (i.e., right-handed individuals associate positive valence to objects in rightward space) (Casasanto, 2009). It is intriguing to speculate that LHb functional/structural asymmetry may contribute to this behavior. While a recent meta-analysis did not find handedness to be a risk factor for depression (Packheiser et al., 2021), hemispheric differences in the treatment of depression using transcranial magnetic stimulation (TMS) have been observed (Chung et al., 2015) suggesting that LHb streamline asymmetry may be of therapeutic importance as well.

After exploring hemispheric asymmetry, we next investigated sex differences in LHb streamline number. We found that women have a greater number of SM/FR streamlines on average compared to men. Our group has previously demonstrated that significant structural and functional differences exist between the male and female human brain (Ingalhalikar et al., 2014; Tunç et al., 2016). The current study supports and extends these findings by revealing increased LHb afferent/efferent streamline count in females compared to males. Female sex is a well-known risk factor for depression and TRD (Taipale et al., 2020). Given the LHb’s role in processing stimuli of negative valence, it is intriguing to speculate that increased LHb streamline number may contribute to susceptibility to depression. One limitation of this study is that the streamlines were not reconstructed in individuals with a diagnosis of major depressive disorder. Future studies could more thoroughly explore the relationship between sex, LHb streamline count, and depression.

## Conclusion

Using the input and output brain regions of the LHb, we have developed a method to reconstruct the afferent and efferent tracts of this epithalamic nucleus without having to identify it manually, which is challenging and error prone due to its small size. This technique enables surgical targeting of the SM and FR tracts as well as indirect LHb targeting. Furthermore, we have demonstrated that these streamlines are more prominent in the female human brain and that most right-handed individuals have a greater number of streamlines in the left hemisphere. These findings may have therapeutic implications for treating affective disorders such as TRD and warrant further investigation in larger studies that have clinical measures for correlation.

## Data Availability Statement

The raw data supporting the conclusions of this article will be made available by the authors, without undue reservation.

## Ethics Statement

Ethical review and approval was not required for the study on human participants in accordance with the local legislation and institutional requirements. Written informed consent for participation was not required for this study in accordance with the national legislation and the institutional requirements.

## Author Contributions

FH: conceptualization, methodology, formal analysis, writing – original draft, and visualization. DP: resources and data curation. AY: writing – review and editing and conceptualization. SB: writing – review and editing, supervision, and funding acquisition. RV: conceptualization, writing – review and editing, supervision, and funding acquisition. All authors contributed to the article and approved the submitted version.

## Conflict of Interest

The authors declare that the research was conducted in the absence of any commercial or financial relationships that could be construed as a potential conflict of interest.

## Publisher’s Note

All claims expressed in this article are solely those of the authors and do not necessarily represent those of their affiliated organizations, or those of the publisher, the editors and the reviewers. Any product that may be evaluated in this article, or claim that may be made by its manufacturer, is not guaranteed or endorsed by the publisher.
