# Supplementary material for: Laterality and Sex Differences of Human Lateral Habenula Afferent and Efferent Fiber Tracts
Source: Front Neurosci. 2022 Jun 16;16:837624. doi: 10.3389/fnins.2022.837624 (PMC9243380; doi:10.3389/fnins.2022.837624)
Supplement: Supplementary file 1 [file Data_Sheet_1.docx]

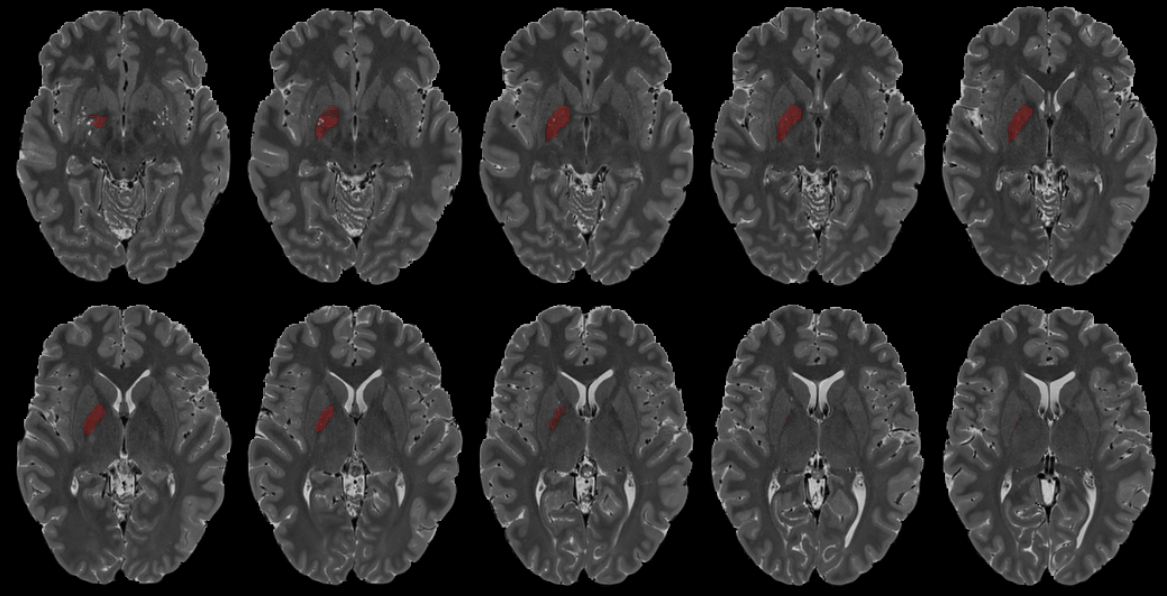


Supplementary Figure 1. Region of interest (ROI) for probabilistic tractography seeding.

FreeSurfer subcortical segmentation was used to obtain the pallidum as a seed region for the tractography. The right pallidum ROI is shown above in red in one subject on axial T2 MRI slices.


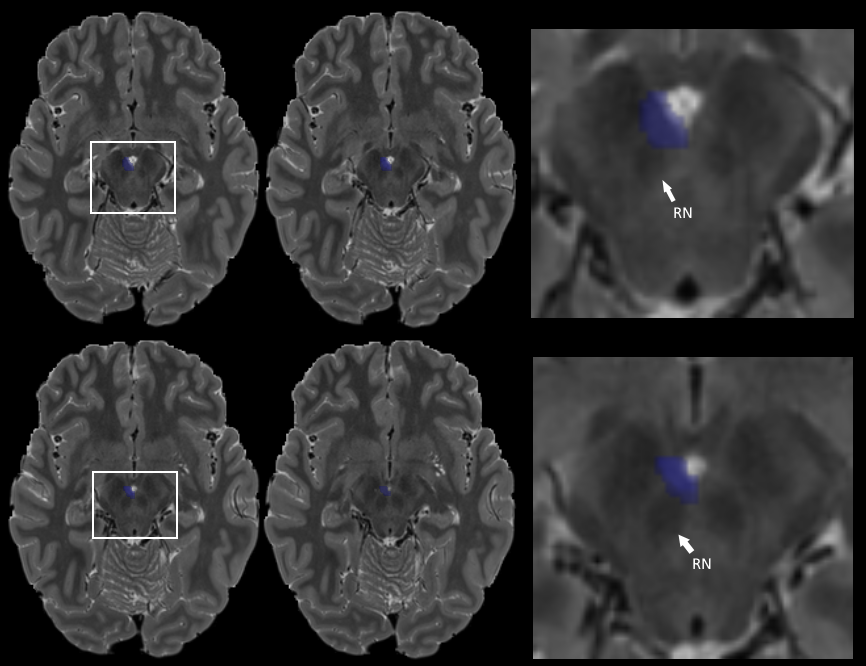


Supplementary Figure 2. Termination mask for probabilistic tractography.

The ventral tegmental area (VTA) was used as the termination mask. This ROI is shown above in blue in one subject on axial T2 MRI slices. The rightmost panels are magnifications of the areas outlined with the white boxes on the images to the left. The red nucleus (RN) is denoted above.


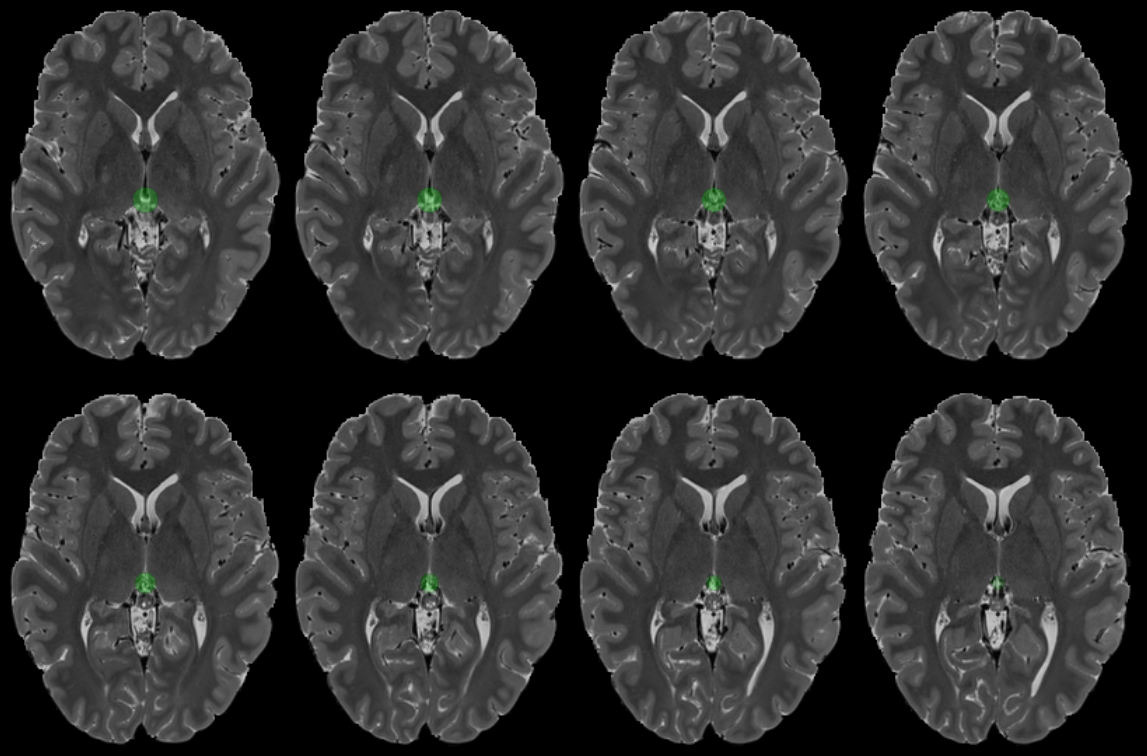


Supplementary Figure 3. Waypoint mask for probabilistic tractography.

The rostral half of a 6mm radius sphere centered at the posterior commissure was selected as the waypoint ROI. This ROI is shown above in green in one subject on axial T2 MRI slices.
